# Report of a Complicated Case of Couvelaire Uterus

**DOI:** 10.1155/2023/6668328

**Published:** 2023-06-27

**Authors:** Hamideh Parsapour, Neda Shafie, Amir Mohammad Salehi, Zeinab Assareh

**Affiliations:** ^1^Clinical Research Development Unit of Fatemieh Hospital, Department of Gynecology, Hamadan University of Medical Sciences, Hamadan, Iran; ^2^Student Research Committee, Hamadan University of Medical Sciences, School of Medicine, Hamadan, Iran

## Abstract

Couvelaire uterus (CU) is a rare complication in the life-threatening placental abruption (PA) that consists of a state of blood infiltration of the uterine myometrium and serosa. The incidence is around 1% and the treatment of choice is obstetric hysterectomy, however, in some cases, close monitoring and timely decision-making can prevent hysterectomy. Herein, we present a rare and serious case of CU with uterus preservation in a young multiparous with a high-risk pregnancy.

## 1. Introduction

Couvelaire uterus (CU) or uteroplacental apoplexy was first described in 1911 by Dr. Alexandre Couvelaire. CU is one of the rare nonfetal complications of severe abruption and is associated with placental abruption (PA); the incidence and maternal mortality are around 1% and 5% [1, 2].

CU manifests when a ruptured decidual spiral artery causes hemorrhage and bleeds into the decidua basalis and myometrium, followed by blood penetration into the uterine serous layer causing a blue-violet ecchymosis [3]. A hysterectomy may be the only way as a life-saving measure if hemostasis cannot be achieved adequately in view of disseminated intravascular coagulopathy. However, in some rare cases, the uterus can be preserved with unstable hemodynamics [4]. Herein, we present a rare and serious case of CU with uterus preservation in a young multiparous with a high-risk pregnancy.

## 2. Case Presentation

Our case report describes a 28-year-old female patient (gravida two, para one) with monochorionic diamniotic twin Gestation, in the 30th week (30 + 6) who presented to Fatemieh Hospital (Hamadan) Emergency with hypertension (blood pressure 140/90) and was hospitalized with a diagnosis of preeclampsia, other vital signs of the patient were normal at the time of admission to the emergency room and the nonstress test (NST) of the twins was reactive. The patient did not report any underlying disease other than chronic hypertension for which no drug was used. She did not mention her history of smoking, alcohol, or drugs.

**Figure 1:**
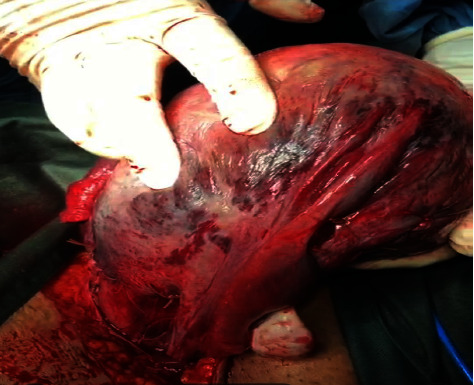
The dark purple and copper color patches with ecchymosis and indurations diagnostic of couvelaire uterus or uteroplacental apoplexy.

The morning after admission, the patient suddenly experienced sweating, anxiety, pain, and tetanic uterine contractions without vaginal bleeding. On examination, the patient had a heart rate 100 per minute, blood pressure 90/70, and respiratory rate 20 per minute. On suspicion of PA, the patient was asked for an obstetric ultrasound, and according to the diagnosis of PA and fetal bradycardia (90–100 beat/min), the patient was immediately transferred to the operating room for cesarean section (CS). The abdominal layers were opened with a Pfannenstiel incision, Kerr incision was made on the uterus. The first infant with an Apgar score of zero and the second infant with an Apgar score of 3–5, expired despite advanced resuscitation, and the placenta was more than 90% detached. After the placenta was removed, large clots were seen behind it, all of which were removed. The patient's uterus was soft, pasty, blue, and Couvelaire. Diffuse hematomas were also seen with spreading in the pampiniform plexus, broad ligament, and posterior uterus. (Figure 1 Surgery video link (https://uupload.ir/view/vid-20220422-wa0026_q6n.mp4/)).

Due to continuous bleeding and anuria, transfusion of 3 packed red blood cells, 3 fresh frozen plasma (FFP), and 3 cryoprecipitate (Cry) were started for the patient. Also, to maintain the uterus and control its atony, compressive sutures were applied and oxytocin (80 U/L IV), misoprostol (1000 *μ*gr rectal), and tranexamic acid (1 gr IV) were prescribed to the patient. After controlling the bleeding, the uterus was returned to the abdomen and a drain was inserted into the patient. According to the goal of fluid and electrolyte correction and due to the continuation of anuria (Creatinine = 2.8), a central venous line was inserted for the patient and a high-dose diuretic (Lasix), labetalol, serum nitro-glycerine, and hydralazine were prescribed to control the patient's accelerated hypertension, the patient's echocardiography was normal; therefore, the patient was transferred to the intensive care unit (ICU) and hospitalized in the ICU for two weeks.

During ICU admission, due to increased creatinine and oliguria, the patient underwent 9-time hemodialysis and due to disseminated intravascular coagulation (DIC), the patient received 11 pack cells, 35 platelets, 20 FFP, 8 Cry, IVIG [1], and dexamethasone (20 mg IV 2 day). Also, due to the possibility of sepsis, vancomycin (1 gr IV 48 h after dialysis) and metronidazole (500 mg daily) were administered GFR adjust and meropenem (500 mg daily). Also, due to the positive culture for acinetobacter, colistin was administered for one week. Anticoagulant (heparin) was prescribed for the patient after the platelet count increased to more than 50,000 (Table 1).

After two weeks, with a gradual decrease in creatinine and improvement of tests, hemodialysis and antibiotics were stopped and the patient was transferred to the ward, and after a week, the patient was discharged from the hospital with Cr = 1/3.

## 3. Discussion

We present an unusual case of a CU that despite unstable homeostasis and severe bleeding in the myometrium and subserosal, without the need for hysterectomy, the patient was successfully treated. CU was associated in different reports with PA, placenta previa, amniotic fluid embolism, and preeclampsia [1]. In our patient, CU was associated with preeclampsia and PA.

The complete or partial separation of site implanted placental before delivery is defined as PA. It happens in 0.8 to 1% of births [5]. The etiology of PA remains unclear. Women with PA are at increased risks of perinatal morbidity and mortality, maternal postpartum hemorrhage, shock, and cardiovascular disease [6]. Maternal asthma, prior CS, cocaine use, endometriosis, chronic hypertension, advanced maternal age, maternal smoking, and use of assisted reproductive technology, obesity, preeclampsia, uterine leiomyoma, and marijuana use were risk factors associated with PA [7]. In our patient, chronic hypertension and obesity that increases her risk of PA.

There is currently no established diagnostic clinical criterion for PA. The most common reason for a clinical diagnosis of abruption, according to the New Jersey-Placental Abruption study [8], was retroplacental clot(s) or bleeding (77.1%), followed by vaginal bleeding with uterine hypertonicity (27.8%) and vaginal bleeding with nonreassuring fetal status (16.1%). In our patient, no vaginal bleeding was observed, but the uterus was extremely hypertonic and the status of the fetuses was uncertain.

The management of PA with CU depends on the patient's status. There have been cases of successful conservative management of CU [9]. The uterine ability to contract is preserved, particularly after amniotomy and decompression to allow constriction of spiral arteries to achieve hemostasis, however, the CU sometimes loses its contractile power and if there is no response to oxytocin and other uterine contractions' stimulant drugs, or acute abdomen and coagulopathy, the patient should be taken to the operating room immediately [1, 4].

The surgical management of the Couvelaire uterus is by hysterotomy or hysterectomy. Hysterectomy can be considered when there is profound myometrial damage or uncontrolled bleeding despite conservative measures by hemostatic brace sutures or uterotonics [4]. In our patient, despite severe PA, the uterus was preserved with a control of bleeding.

## 4. Conclusion

The unpredictability of this clinical condition and the importance of close vigilance and timely decision-making can prevent adverse maternal and fetal outcomes.

## Figures and Tables

**Table 1:** Laboratory test.

Test	Admission	In the operating room	The first day of hospitalization in the ICU	The 4th day of hospitalization in the ICU	The 7th day of hospitalization in the ICU	The last day of hospitalization in the ICU	The day of discharge from the hospital	Unit of measurement
WBC	7500	17200	20000	9900	12000	10000	6600	Mg/dl
Hb	13	11.9	8.9	8.1	9.3	8.9	9.6	Mg/dl
Platelet	155000	118000	36000	44000	50000	95000	231000	Mg/dl
Cr	0.9	1.23	2.84	5.2	6.8	2	1.3	Mg/dl
AST	10	10	85	205	180	40	—	u/l
ALT	5	5	100	220	110	14	—	u/l
LDH	400	718	1686	3760	958	3760	200	Mg/dl
PT	—	18	14	14	15	15	12.5	Second
PTT	—	37.7	48	45	42	37	34	Second
INR	—	1.4	1.3	1.3	1.2	1.3	1.3	
Fibrinogen	NA	84	210	139	185	220	230	Mg/dl
D-dimer	—	2568	—	—	1987	—	400	Ng/dl
Urine protein	++	NA	+	—	—	—	—	Mg/dl

## Data Availability

The data used to support the findings of the study are available from the corresponding author upon request.

## Abbreviations

CU: Couvelaire uterus
PA: Placental abruption
NST: Nonstress test
CS: Cesarean section
FFP: Fresh frozen plasma
Cry: Cryoprecipitate
DIC: Disseminated intravascular coagulation
ICU: Intensive care unit.
